# Through Time and Across Traits: Evaluating Explanations of Human Evolution

**DOI:** 10.1002/evan.70046

**Published:** 2026-08-20

**Authors:** Miki Ben‐Dor, Ran Barkai

**Affiliations:** ^1^ Department of Archaeology Tel Aviv University Tel Aviv Israel

**Keywords:** directional evolution, explanatory completeness, hominin, human evolution, niche construction theory, prey‐size decline, threshold and gradient causes, Tinbergen's four questions

## Abstract

We argue that current explanatory practice misses two situations that can arise in any lineage, not only ours. The first is dynamic: traits continue changing in one direction when a lineage's own niche construction keeps enlarging the problem it faces. The second is unifying: many traits change together and may require one explanation rather than several. Both cases bear on Tinbergen's four questions, the test of explanatory completeness, and both require extending his function question: what is a trait for? An answer that names a problem of fixed size cannot explain continued change. We call a problem of rising magnitude a gradient, and one that serves many traits a unifying gradient. We set out five questions that any complete gradient explanation must answer, map the major hypotheses of human evolution onto this scheme, and use our Prey‐Size Decline hypothesis as a worked example. The proposed framework does not depend on that example.

## 1 Introduction

Human evolution lacks a widely accepted unifying explanation. It need not have one. Biological history need not resolve into a single organizing principle in the way geology is resolved into plate tectonics, and a field can be in good order without a theory of that form. What the field lacks is an agreed way of stating what kind of question a given hypothesis answers. Explanations of human evolution are numerous and often not in direct competition, because they address different questions.

Tinbergen's scheme already supplies that discipline. A complete biological explanation must answer four logically distinct questions: how a trait is produced, how it develops in the individual, what selective problem it solves, and how it evolved from ancestral states [1]. The scheme need not be replaced or supplemented with a fifth question. One of the four, the function question, is conventionally asked in a way that cannot detect two kinds of incompleteness in the answers it returns, and both matter for any lineage showing sustained directional change.

Asked once of a single trait, the function question yields a still photograph, this is the trait, and this is the problem it answers. Where the problem does not change across generations, a still is enough. A lineage that changed across several traits for two million years requires motion as well as focus: the question must be asked through time, and the answers compared across traits.

The first addition is temporal. Asked repeatedly of a trait that continued to change, the function question either returns a different problem at each step, in which case the trajectory is a series of thresholds requiring an account of why they aligned, or the same problem at rising magnitude, in which case the problem itself grew. A gradient is therefore a selective problem whose magnitude rises because the response to it helps generate the next problem. The second addition is cross‐trait. Tinbergen's scheme is applied trait by trait and is silent on whether the answers given for different traits are one answer or several; in a lineage showing co‐directional change across many traits, that difference is the difference between a single explanation and a coincidence of separate ones.

The additions are motivated by the human record, which appears to show sustained directional change across several traits. That claim has been contested, most directly by Gould and Eldredge [2], and more recently by those who emphasize the mosaic and episodic character of the record and the documented decline in endocranial volume since the terminal Pleistocene [3, 4]. Directionality must therefore be defined before the record is interpreted, and the same standard must govern candidate explanations. Otherwise, an explanation built to explain a pattern can always be accused of having defined the pattern to suit itself.

## 2 The Shape of the Human Trajectory

### 2.1 What Would Count as Directionality

Fossil time series can be treated as sequences of steps between successive intervals [5]. Two independent quantities describe such a sequence: the mean step, which determines whether the trait is displaced over the long run, and the variance of the steps, which determines how violently it moves. A series is directional over an interval when the mean of its steps differs from zero, tested by asking whether a directional model fits better than an unbiased random walk or than stasis. Volatility is irrelevant to this distinction: a series with large canceling steps is not directional, whereas a series with small consistently biased steps is.

Directionality in this sense is distinct from monotonicity. Monotonicity is the degree to which change runs with the trend rather than against it, and it is measured rather than assumed, most simply by Kendall's tau, which is zero for a series with no trend and ±1 for a strictly monotonic one, positive for an increasing series and negative for a decreasing one. The two are governed by the ratio of the mean step to its variance. A driver whose mean step is small relative to the variance produces a series resembling noise at fine temporal grain and a trend at coarse grain, with a calculable frequency of reversals. Scale dependence of this kind is a prediction of a weak but persistent directional pressure rather than evidence against one, and it is what Du et al. [6] report for hominin brain size, where a gradual clade‐level trend resolves at finer scale into episodes of directional change interspersed with stasis and drift.

These definitions set the empirical stakes. If hominin series are best fitted by unbiased random walks, there is no sustained directionality to explain. If they are strictly monotonic, a model in which directional pressure operates alongside oscillating ones fails in a different way. The expected pattern is directional but only moderately monotonic: net displacement over the interval, with reversals and plateaus at finer temporal grain.

### 2.2 What Would Count as Complexity

Because complexity is used loosely in this literature and can imply progress, the term is restricted here to four independently measurable quantities, none of which ranks cultures by worth. Technological complexity comprises procedural‐unit count, the mutually exclusive manufacturing steps contributing to the finished form [7], with a coded 3.3 Myr dataset available in Paige and Perreault [8]; techno‐unit count, the separate physical components combined in a working implement [9], which distinguishes a handaxe from a hafted composite projectile; raw‐material selectivity, measured by the shift from secondary to primary sources and by transport distance [10, 11]; and mean worked‐element size.

Declining element size is causal rather than definitional. Small elements function only as parts of multi‐component systems, so miniaturization and rising techno‐unit counts are two aspects of the same shift to composite technology. Toolkit diversity is likewise not equivalent to adaptive complexity: diversity refers only to assemblage richness.

### 2.3 The Traits Considered, and the Level at Which Change Occurred

A claim that several trait classes changed in a common direction is informative only if the candidate set is stated. The relevant traits are brain volume, the four technological measures above, foraging range, life‐history schedule, cooperative organization, body size, postcanine tooth size, and sexual size dimorphism. Brain volume, the technological measures and foraging range can be plotted against time with usable resolution. Life‐history schedule is represented by a handful of specimens with dental development data and cannot be plotted. Cooperative organization has no accepted quantitative proxy and is treated as functionally entailed rather than independently measured, making it the weakest link in the argument. Postcanine tooth size reduces through the lineage but does not track brain enlargement [12] and therefore fails to co‐vary.

The level at which change occurred is separate from whether it occurred. Apparent directionality in a clade average can be produced by differential survival among lineages rather than by change within them, and the two are not causally equivalent. Gould and Eldredge [2] stated the alternative for this case specifically: no gradualism, in their reading, had been detected within any hominid taxon, and the trend to larger brains arises from the differential success of essentially static taxa. If so, the directionality at issue is a property of clade composition rather than selection operating within lineages, and an account of sustained selective pressure would be misplaced.

Recent quantitative work supports the within‐lineage reading. Du et al. [6] partition the hominin endocranial trend and find 64%–88% of it generated within hypothesized lineages rather than by species turnover, depending on whether a more or less speciose taxonomy is adopted. Püschel et al. [13], applying a phylogenetic approach not previously used on paleoanthropological data, likewise find that relative brain‐size increase arose from increases within individual species. The two levels are not rivals: a pressure acting on every lineage that engages a given niche predicts directional change within each lineage that tracks it and differential persistence among those that do so less well. They are consequences of one cause, and an account yielding only one of them is incomplete.

### 2.4 What the Record Shows

**Figure 1:**
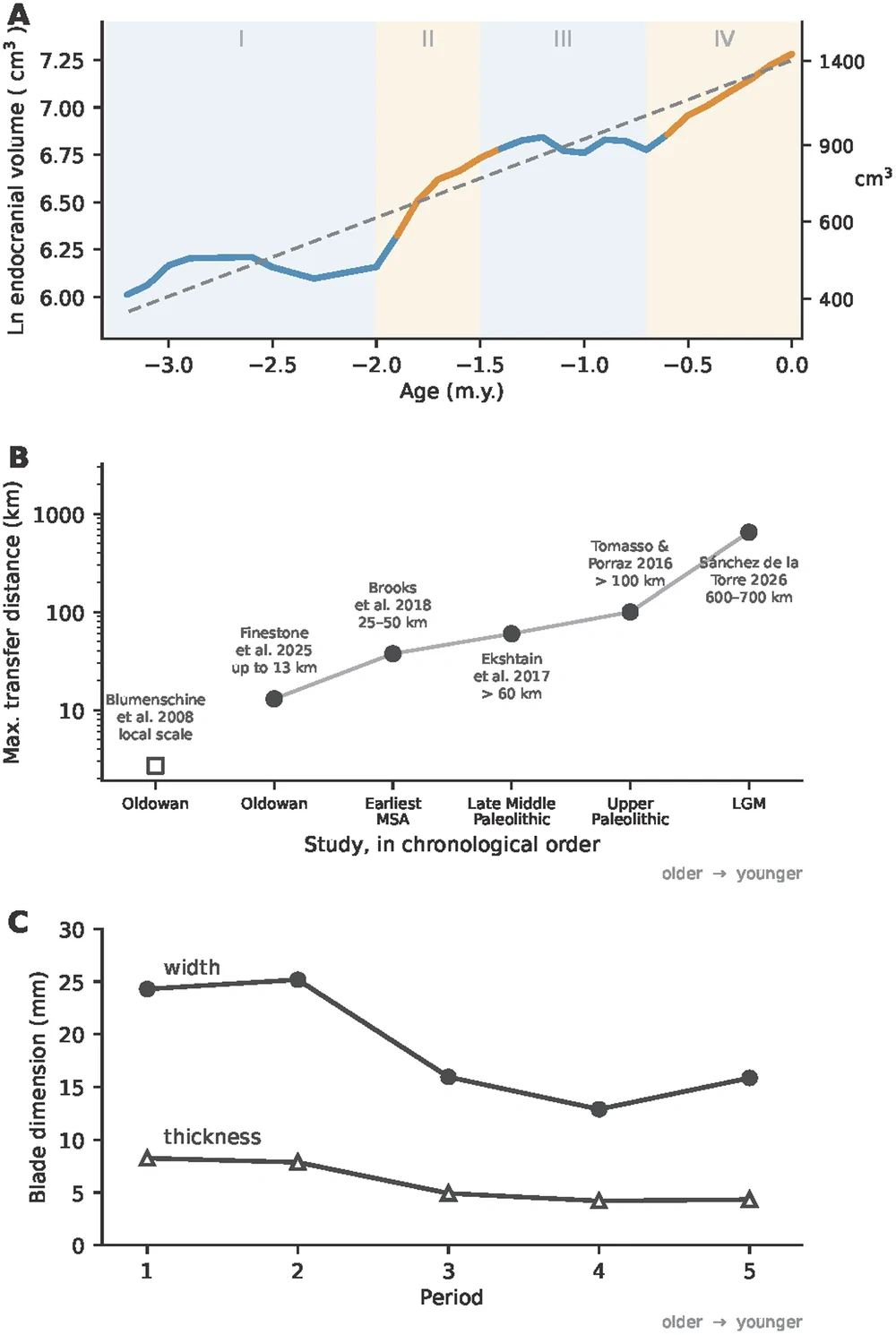
Directional change in three trait classes, each measured independently and plotted on the time scale used by its source. (A) Endocranial volume. The line traces the 1:2:1 weighted running mean of ln endocranial volume in successive 100‐kyr bins, computed from the 233‐specimen consensus series of [14]; 28 bins contain at least one specimen. Segments are colored by the four phases of stasis (blue, I and III) and directional change (amber, II and IV) that Gingerich found statistically distinguishable from random change. The dashed line is the long‐term regression across the series, ln ECV = 0.415 · Age + 7.248. (B) Maximum raw‐material transfer distance in six studies, in chronological order: early Oldowan, Olduvai [15]; Oldowan, Nyayanga [16]; earliest Middle Stone Age [17]; late Middle Paleolithic, Amud Cave [18]; Mediterranean Upper Paleolithic [19]; Last Glacial Maximum, western Europe [20]. Ranges are plotted at their midpoint and values reported as a minimum are plotted at that minimum, so every point lies at or below what its source reports. Blumenschine et al. report distance‐decay at local scale without a maximum and are shown as an open symbol. (C) Mean width and mean thickness of laminar blanks by chronological period, from Khun and Shimelmitz [21], Table 2: unweighted averages over more than 100 assemblages. Their periods are 1, mid‐Middle Pleistocene (Amudian and pre‐Aurignacian, Fauresmith); 2, late Middle Pleistocene and early Upper Pleistocene (Middle Paleolithic, Middle Stone Age); 3, 50–25 ka; 4, 25–12 ka; 5, Holocene. Rank correlations against period are −0.38 for width (*n* = 111) and −0.44 for thickness (*n* = 109), both *p* < 0.01. (B and C) use ordinal rather than calendar axes because the underlying studies and periods do not share a common dating scheme, and precise ages are unavailable for many assemblages in (C).

Quantitative data exist for three trait classes: endocranial volume, maximum raw‐material transfer distance, and lithic element size. Endocranial volume and element size come from published compilations, which we plot but did not assemble; the transfer‐distance values we assembled ourselves from separate studies. None of the published studies were carried out with the argument made here in view. Endocranial volume is the best‐resolved series and the only one for which model fits of the kind described in 1.1 have been published: Gingerich [14] identifies four phases significantly different from random, alternating stasis with directional increase, the first increase beginning at 2.0 Ma (Figure 1A). Absolute volume roughly doubles between early *Homo* and late *Homo sapiens*.

Endocranial volume defines the clearest lower bound for the interval. Directional change begins at about 2.0 Ma in the best‐resolved series, and the physiological boundary discussed in Section 3 points to the same interval. The relevant interval is therefore roughly the past two million years rather than the past three.

The technological measures do not move together. Cutting‐edge yield per unit mass of raw material rises sharply from the Oldowan to the Middle Paleolithic and then does not increase significantly [22]. Procedural‐unit counts are close to flat until roughly 600 ka and rise thereafter [23]. Mean element size falls throughout, and most steeply in the interval where the first two are flattening, in both width and thickness, across 111 laminar assemblages spanning the mid‐Middle Pleistocene to the Holocene [21], Figure 1C. Following the ratified definition, the Pliocene–Pleistocene boundary is 2.58 Ma [24], although some of the literature cited here predates ratification and uses the earlier 1.8 Ma convention.

Maximum raw‐material transfer distance rises by roughly two orders of magnitude across the same interval, from the local scale of the early Oldowan to several hundred kilometers by the Last Glacial Maximum (Figure 1B). The series is a maximum rather than a central tendency, so it is sensitive to sample size and to how thoroughly sources have been characterized in each region. Transfer distance is also an imperfect proxy for ranging behavior, since mobility, exchange and differential discard probability can produce similar distance‐to‐source distributions. A third limit follows from how the panel is built. Its points come from separate studies in different regions and periods, so it is a compilation of maxima rather than a time series in the sense of 1.1, and the model comparison described there cannot be run on it. Panel B shows the magnitude of the change and not its mode; a fit would require a regionally consistent series constructed for the purpose. It is therefore one strand among several rather than an independent measure of range.

The informative axis of technological response shifts across the interval, from edge yield to compositeness to miniaturization and delivery, and no single metric tracks the whole of it. That shift is a property of the record rather than a defect of the measures. It also constrains acceptable explanations: a candidate that predicts which technological axis should be under pressure at which time is more testable than one predicting a general rise in complexity. The panels are accordingly not expected to move in step. They rest on different time bases, and response systems with different costs, constraints and lags will change at different times even under a single pressure; tight correlation among them would suggest one response rather than several. What the figure shows is a common sign across three independently measured trait classes, not a synchronized trajectory.

The rank‐order correlations reported for blade dimensions show that those dimensions decline monotonically against ordinal period at conventional significance; they do not discriminate among the modes set out in 1.1, because a rank‐order correlation against an ordinal series tests the sign of a trend rather than the step‐to‐step structure by which a directional walk is distinguished from an unbiased one. A gradient cause must explain directional mode sustained over a long interval, not monotonicity as such. Endocranial volume remains the only series for which that discrimination has been performed [6, 13, 14], and the technological series are consistent with that pattern but do not yet meet the central empirical demand.

### 2.5 The Established Pattern and Its Limits

What the record supports is a set of partly correlated directional episodes sharing a common sign across roughly two million years, distributed across a branching clade in which species overlapped, diverged, contracted, exchanged genes and went extinct. It is not a single continuous monotonic process running through one population. Individual traits follow mosaic paths and some reverse locally; endocranial volume in *Homo sapiens* has declined since the terminal Pleistocene [4]. Two of the trait classes we would like to include cannot be measured on a common scale at all, and one trait we examined moves against the trend.

The explanandum is therefore conservative. It does not require a smooth, lineage‐wide, or synchronous trajectory across traits, and it remains meaningful even if the pattern is weaker than strong directional accounts have claimed. A framework that depended on perfect monotonicity or uniform trait covariance would fail against the mosaic and episodic character of the record.

Even in this conservative form, the pattern is unusual. Fitting stasis, unbiased random walk and directional models to 251 trait sequences from 53 lineages, Hunt [25] found directional change the best‐supported mode in 5% of cases; more sensitive methods raise this to 13% [26]. Because researchers have preferentially studied lineages with prior evidence of gradual change, the sample is likely enriched for directionality. Hunt attributes the rarity to directional episodes being either infrequent or too brief to resolve, below roughly 10,000 years in most depositional environments. The hominin trajectory is therefore unusual less for being directional than for the duration over which directionality was sustained, which exceeds that ceiling by two orders of magnitude. Duration is the feature requiring explanation.

Two qualifications follow. Not every trait in the hominin package requires a directional cause: some are plausibly exaptations, some are developmental consequences of change elsewhere, and some are responses to several pressures at once. The threshold and feedback hypotheses are also not mutually exclusive. The framework classifies them rather than selecting one in advance.

## 3 Two Additions to the Function Question

### 3.1 What Tinbergen's Questions Are Asked Of

The empirical limits just described determine what the conceptual framework must do. It must preserve the modesty of the record while distinguishing ordinary stabilization, one‐off transitions, and sustained directional pressure. Tinbergen's function question supplies the starting point because it already asks what selective problem a trait solves; the missing distinction concerns how often that answer must be given, and whether the same answer recurs across traits. Section 2 distinguishes threshold from gradient answers; Section 3 states what a gradient must explain; Section asks which existing hypotheses can meet those requirements. Tinbergen [1] argued that a complete biological explanation must answer four logically distinct questions: mechanism, ontogeny, function and evolution. His central methodological point was that the four are complementary rather than competing, and many apparent disagreements dissolve once the disputants recognize that they are answering different questions about the same trait. Mayr [27] collapsed these into the proximate and ultimate distinction, but the four‐fold scheme remains the standard analytical tool in evolutionary anthropology and behavioral ecology.

Recent applications show why that discipline matters for human evolution. Vinicius et al. [28] apply Tinbergen's scheme to human cumulative culture and reach the same diagnosis of the field's habits: models of brain expansion “often treated as alternatives are in fact complementary to each other.” Their assignment of the technological hypothesis to adaptive explanation, the expensive brain to mechanistic explanation and heterochrony to ontogenetic explanation shows why apparent rivalry often reflects answers to different questions. Their Foraging Niche Hypothesis then occupies all four questions in turn.

That account also exposes a division inside ultimate explanation. One component is a foraging niche whose occupation created selective pressure for lithic dependence and long‐range mobility, initiating a transition. The other is a cultural ratchet in which “the main selective pressure driving human cognitive evolution would be imposed by a growing body of [cumulative culture],” a quantity that grows. These are answers of different kinds, with different evidential burdens, even though both are called ultimate. Table 1 accordingly enters their work in two rows, once in each subcategory, which is not an inconsistency in the table but the clearest single illustration of why the subcategories are needed.

**Table 1:** Classification of the major hypotheses of human evolution by Tinbergen's four questions, with the function question divided into threshold and gradient subcategories.

Hypothesis	Mechanism	Ontogeny	Function: Threshold	Function: Gradient	Phylogeny
Expensive tissue hypothesis [29]	P				
Expensive brain framework [30]	P				
Cooking hypothesis [31, 32]	P		S		
Endurance‐running hypothesis [33]	P		S		
Cognitive‐niche hypothesis [34]	P		S		
Foraging niche hypothesis [28, 35]†	P		S		
Human‐niche hypothesis [36, 37]	P			C	
Learning‐mechanism feedback hypothesis [38]	P			C	
Cumulative culture and social ratcheting [28, 39, 40]†	P			C	
Cultural brain hypothesis [41]	P	S		C	
Social brain hypothesis [42, 43, 44]	S			C	
Machiavellian‐intelligence hypothesis [45]	S			C	
Social‐competition hypothesis [46]	S			C	
Sexual‐selection/mating‐mind hypothesis [47]	S			C	
Embodied capital theory [48]		P	S	C	
Grandmother hypothesis [49]		P	S		
Provisioning/pair‐bonding hypothesis [50]		S	P		
Savanna hypothesis [51, 52, 53]			P		
Turnover pulse hypothesis [54]			P		
Hunting hypothesis/man the hunter [55, 56]	S		P		
Genetic/neural mutation hypothesis [57]	S		P		
Self‐domestication hypothesis [58, 59]	S		S	C	
Variability selection hypothesis [60, 61]				C	
Phylogenetic comparative work [62, 63]					P
Prey‐size decline [64, 65]			S	P	

*Note:* Key: P = primary slot; S = secondary or partial contribution; C = candidate gradient; blank = not addressed. A single publication may support more than one hypothesis and therefore appear in more than one row; † marks the case discussed in Section 2.1.

The evolutionary half of Tinbergen's scheme is the relevant domain. Mechanism and ontogeny are not questions about the evolution of a trait. Tinbergen's questions are asked of traits, whereas the pattern at issue is a trajectory across several traits. Asking his questions of a trajectory would be a category error, since a trajectory is not the sort of thing that has a function. The questions must be asked of traits one at a time; the trajectory emerges when the answers stand in a particular relation to one another.

Two of the trait classes are archeological rather than anatomical. A stone tool is not a trait of an organism in the narrow sense, but lithic attributes can be treated as extended phenotypic products [66]. Foraging range, life‐history schedule and cooperative organization are traits in the ordinary sense.

The function question is not asked of a trait in isolation. Tinbergen's own statement of it is relational: one wants to know “whether a bill of this size and this shape is best suited to feeding in the environment in which the Blackbird lives” (Tinbergen [1, p. 419]). The question is comparative and quantitative in form (this size and this shape rather than some other) and it is asked against a specified environment. An environment is therefore not an external condition under which the function question happens to be answered. It is one of the question's terms.

The evolution question, as Tinbergen posed it, is also larger than the use now made of it. “Evolutionary study has, of course, two major aims: the elucidation of the course evolution must be assumed to have taken, and the unraveling of its dynamics” (Tinbergen [1, p. 428]). The first aim is reconstruction of descent. The second he divides into the genetic control of species‐specific behavior and “the study of the influence of selection on behavior evolution.” Modern restatements of the four questions render this cell as phylogeny and leave it there. The second aim, which is where a driving selective pressure would be stated, has largely dropped out of the scheme as the scheme is now used.

It matters that Tinbergen was explicit about what the loss costs, because the distinction he drew is the one this paper turns on. A demonstration of survival value, he says, establishes one thing firmly: “one can say that one has demonstrated beyond doubt a selection pressure which prevents the species in its present state from deviating. One has really demonstrated the part played by selection in stabilization of the present state. However, the conclusion that this same selection pressure must have been responsible in the past for the molding of the character studied is speculative, however probable it often is” (Tinbergen [1, pp. 428–429]). Stabilization is what a function answer demonstrates. Molding is what is habitually taken to show. The inference from the first to the second is not licensed by the demonstration.

Tinbergen then names the condition under which the inference can nonetheless be supported. One may marshal supplementary evidence, “such as arguing that the environment which exerts the selection pressure may be assumed to have remained constant in this respect for a long time or showing that the species is even now slightly variable according to area” (Tinbergen [1, p. 429]). The license for reading a molding causes off a stabilizing function is an assumption of environmental constancy. Where that assumption holds, the practice of asking the function question once is not merely convenient but correct, and this is the condition Tinbergen's own examples satisfy. Where it fails, the license is void, and his own chosen illustration shows what failure looks like: the ultimate test of survival value, he writes, is Kettlewell's work on *Biston betularia* “in two different environments: one which favored the white form, and one which favored the dark form” (Tinbergen [1, p. 423]). One trait, two environments, opposite answers, and the moth unchanged throughout.

This also explains why the loss went unnoticed for so long. Under the constancy condition, the driver is not recovered from the function answer; it is identical to it. If the environment has not changed, the pressure holding a trait where it is simply is the pressure that built it, and stabilization and molding coincide. Where a single transition intervenes, the driver is recovered by subtraction: the function answer gives the pressure after the transition, the evolution question gives the ancestral state before it, and the driver is the difference between them. Constant before, constant after, one step in between, two known endpoints. In neither case does anything remain to be separately stated, which is why a scheme with no cell for a driving pressure has nonetheless served the field for 60 years.

Neither route is available for the problem of rising magnitude. There is no pair of states whose difference is the driver, because the environment confronting each generation is the output of the preceding one. What has to be identified is not a difference but a rule generating a sequence, and a rule cannot be read off any two of its outputs. A problem of rising magnitude is therefore the only case in which the driver is not already implicit in what the other questions answer, and the only case in which it must be asked for in its own right. What is asked for is the rule: what kept the problem growing. That is a question about the dynamics of evolution rather than about its course, and a scheme that renders the question of evolution as phylogeny has left nowhere to record it.

The moving term need not be niche construction. A directional trend in climate would defeat the two recovery routes equally well, and an account of that form would also be a gradient in the sense used here. Niche construction matters because it offers a clear way for an environment to move continuously and directionally rather than fluctuate: the lineage's own activity can make the next generation's problem larger.

### 3.2 First Addition: Asking the Function Question More Than Once

The function question is normally asked once: a trait is observed in its present form, and one asks what selective problem it solves. Under the constancy condition this is adequate. The gull chick faces the same pecking problem its parents faced, and the graylag goose the same displaced egg; the traits that answer such problems are maintained by selection rather than driven by it. These are stabilization cases in Tinbergen's sense, and their abundance explains why asking once has served the field so well.

Two modern amendments make serial answers informative. Bateson and Laland [67] note that fitness now means reproductive success rather than survival alone, so the answer has magnitude; they also prefer current utility to adaptive significance because current and original function can differ. If function has magnitude and can change over time, repeated answers can reveal a trajectory rather than merely restating a verdict.

Applied serially—to early *Homo erectus*, later *Homo erectus*, *Homo heidelbergensis* and *Homo sapiens*—the function question can return one of three answers.

First, the problem may differ at each step. The trajectory is then a series of thresholds requiring separate explanations, plus an account of why those explanations aligned in direction. This is the case Bateson and Laland recognize when characteristics change function over time, as feathers did in passing from thermoregulation to flight.

Second, the same problem may recur at the same magnitude. A larger brain would then confer a fixed advantage while its energetic cost rises with size already attained. A constant benefit set against a non‐falling cost yields an optimum, and selection at an optimum is stabilizing. The constant answer therefore predicts that the trajectory should stop; repeated across successive times, it exposes a contradiction that a single answer conceals.

Third, the same problem may recur at rising magnitude. Meeting it in one generation raises the level at which it must be met in the next, so the trait keeps moving because the marginal return to a further increment does not fall away. We call such a re‐posing problem a gradient.

Serial application distinguishes changing problems, one problem at constant magnitude, and one problem at rising magnitude. Only the third can account for sustained directional change without multiplying independent thresholds, and it creates a further requirement: the rising magnitude must itself be explained. The distinction is generated by Tinbergen's own question rather than imported to supplement it. We are not proposing to replace the four‐question scheme, nor to add a fifth question. We are proposing that one of the four be asked more than once and observing what follows.

Where the environment did not stand still, a function answer must state what problem the trait solved, whether the problem or its magnitude changed, and what generated the change.

Several hypotheses already supply endogenous gradients. On the Social Brain Hypothesis, the problem is coalition tracking in a group whose size increases because the capacity did. On Embodied Capital Theory, the level of skill worth acquiring rises as the period available for acquiring it lengthens. Such accounts differ in what carries the rising magnitude of the problem.

**Figure 2:**
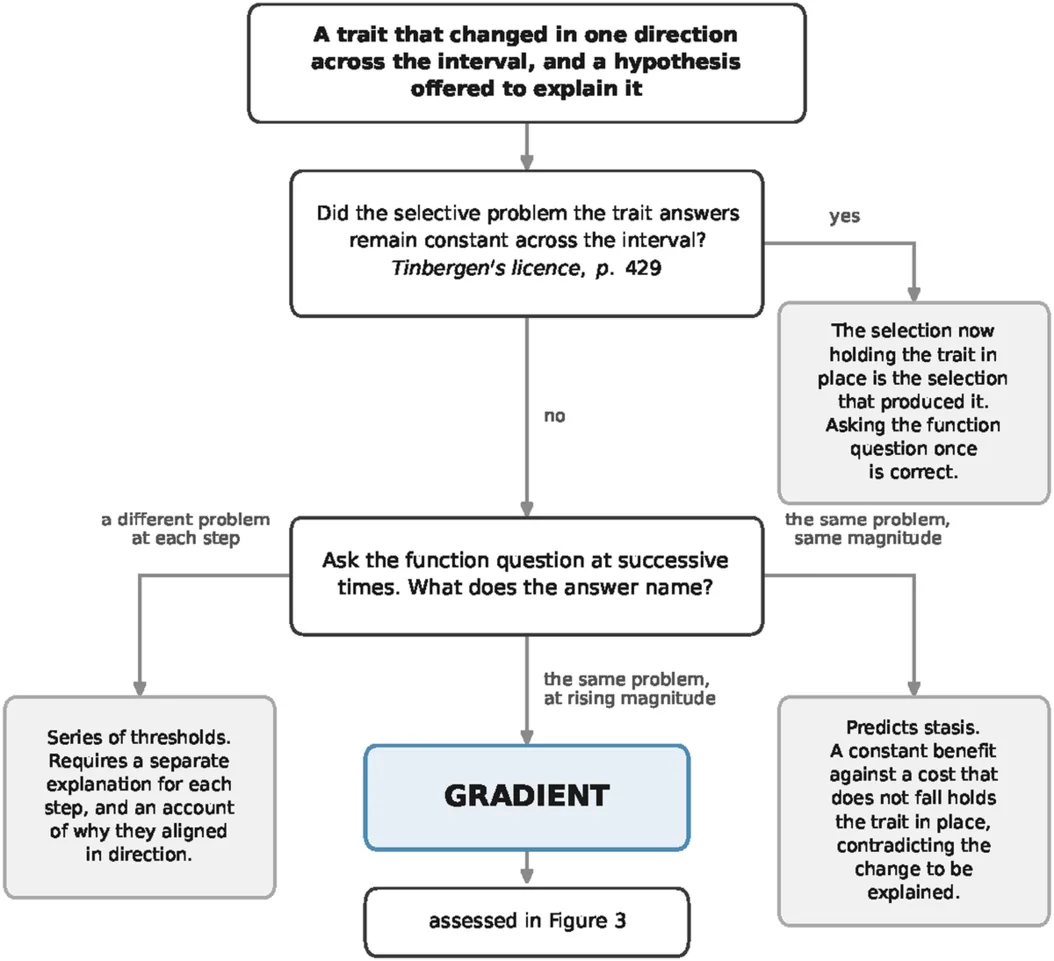
Routing a candidate explanation. The routing logic follows from the constancy condition. If the selective problem remains constant, present function can be read as past molding pressure. If it did not, successive function answers distinguish changing problems, one problem at constant magnitude, and one problem at rising magnitude. A gradient verdict then requires the five questions in Figure 3.

The gradient category has remained largely unoccupied because a question asked once cannot reveal that its answer must be re‐given, and a version of Tinbergen's scheme reduced to descent offers nowhere to record such an answer. The savanna hypothesis, the Turnover Pulse Hypothesis, the cooking hypothesis and entry‐into‐a‐foraging‐niche accounts are therefore not defective; they are answers to a question asked once, in a field without a vocabulary for noticing that it needed to be asked again (Figure 2).

### 3.3 Second Addition: Asking Whether One Answer Serves Many Traits

After serial application, one problem remains: the unit of explanation is still a single trait. A re‐posing problem for one trait does not show whether several changing traits face the same re‐posing problem. The next incompleteness appears only when the explanation shifts from one trait to a package of traits.

Tinbergen's scheme is applied trait by trait, and it is silent on the relation between the answers given for different traits. That silence is unproblematic for a single behavior in a stable environment. It becomes a gap for a lineage in which many traits changed together in a common direction, because the scheme provides no way to ask whether the function answers for those traits are one answer or several.

The question matters because the two possibilities are different explanations. If each of the hominin traits faced a distinct intensifying problem, the trajectory is a set of separate gradients that happened to coincide in direction, and each requires its own account together with an account of the coincidence. If one problem is the answer for all of them, the trajectory has a single explanation. Nothing in the first addition settles this. A trait‐by‐trait application of the threshold and gradient distinction can be carried out completely and still leave the package unexplained as a package.

A lineage showing co‐directional change across trait classes therefore requires a cross‐trait version of the function question. A candidate answer must be assessed not only for each trait separately but for how many traits it serves. An answer that serves many of them is a unifying gradient. The claim that one gradient covers the hominin package is a parsimony claim: a problem that plausibly demands several traits at once is preferable to several problems that must be separately established and then separately shown to have aligned.

The two additions do different work. The first concerns the structure of evolutionary explanation and can be assessed independently of any particular hypothesis about human evolution. The second makes the account explanatory for a trajectory rather than merely diagnostic for a trait. One can accept the first while rejecting any proposed occupant of the second.

Both additions are general. They would arise in any lineage that changed directionally across a long interval, including coevolutionary arms races, host‐parasite systems, domestication, cumulative cultural systems and long‐running ecological engineering. Human evolution is a favorable test case because the record is dense enough to make the distinctions operational and the literature large enough to make the classification informative.

### 3.4 The Kind of Cause a Gradient Requires

A gradient requires a cause capable of producing a selective problem that re‐poses itself in intensified form. Niche Construction Theory supplies that structure.

Niche Construction Theory [68] treats organism‐environment interaction as a feedback loop rather than as a one‐way selective arrow. Organisms inherit modified environments as well as genes, and those modified environments alter the selection their descendants face. The theory has been embedded in the Extended Evolutionary Synthesis [69] and applied from beaver dams to gut microbiomes [70]. For human evolution, Smith [71, 72] characterized humans as the ultimate ecosystem engineers, with hunting, foraging, fire use and ultimately agriculture as the principal niche‐constructing activities [36, 37], and subsequent work has connected NCT to gene‐culture coevolution [73] and to syntheses of human ecological history [74]. A lineage whose own activity progressively transforms the resource base it depends on faces a selective problem that returns in intensified form, which is the structure the first addition calls for.

Niche Construction Theory has been criticized on the ground that its insights are already available in existing concepts such as arms races, runaway selection, ecological legacies and coevolution. The required structural feature is the one both sides accept: selection on a niche‐constructing lineage is partly a function of that lineage's own past activity. The difficulty in the hominin literature is that this structure has not been recognized as supplying a function answer that must be re‐given.

Niche construction also has measurable consequences for technological structure rather than merely redescribing it. Collard et al. [75] examined toolkit diversity and complexity across hunter‐gatherers and small‐scale food producers and found that the variables governing toolkit structure differ systematically between the two, as a niche‐construction reading predicts. The result concerns recent societies rather than the Pleistocene, but it shows that the approach has empirical purchase (Box 1).

Box 1 **Glossary of the hypothesis discussed in this chapter title.**

*Expensive Tissue Hypothesis* [29]. Proposes that the metabolic cost of an enlarged brain was offset by a reduction in gut size made possible by a higher‐quality diet, so that encephalization was energetically enabled rather than directly driven.

*Prey‐size decline hypothesis* [64, 65]. Argues that the progressive Pleistocene decline in the body size of hunted prey imposed sustained selection for technological, cognitive, ranging and cooperative adaptation. It is worked through in Section 3 as a demonstration of the method; the framework does not depend on it.

*Savanna hypothesis* [51, 52, 53]. Attributes the origin of key hominin traits, such as bipedalism and tool use, to the late‐Miocene to Pliocene spread of open grassland, treated as a one‐off environmental transition.

*Cultural‐niche hypothesis* [40]. Holds that the human capacity for high‐fidelity social learning and cumulative culture is the key adaptation, letting populations accumulate locally adaptive knowledge that no individual could invent alone.

*Endurance‐running hypothesis* [33]. Proposes that selection for long‐distance running shaped the genus Homo and supported a hunting and scavenging niche through persistence pursuit and improved access to carcasses.

*Machiavellian‐intelligence hypothesis* [45]. Holds that the cognitive demands of social manipulation, alliance formation and deception within groups drove the evolution of primate and human intelligence.

*Social Brain Hypothesis* [42, 43, 44]. Holds that primate and human brain size is driven primarily by the cognitive demands of maintaining large and socially complex groups, group size, and cognitive capacity increasing together.

*Human‐niche hypothesis* [36, 37]. Frames human evolution through niche construction, cooperation, and imagination, with distributed cognition and the constructed social niche as central drivers.

*Social‐competition hypothesis* [46]. Attributes the elaboration of general cognitive ability to social and ecological competition and to the motivation to control resources and relationships.

*Self‐domestication hypothesis* [58, 59]. Proposes that selection against reactive aggression produced increased tolerance and cooperation, accompanied by a suite of domestication‐like changes in anatomy and behavior.

*Grandmother hypothesis* [49]. Holds that provisioning by post‐reproductive women improved grandchildren's survival, selecting for menopause, extended longevity and the slow human life history.

*Expensive brain framework* [30]. Generalizes the expensive‐tissue idea: the cost of a large brain must be met either by raising total energy intake or by reducing energy allocated to digestion, locomotion, growth or reproduction.

*Embodied capital theory* [48, 78]. Explains the human life‐history package, including a long juvenile period, delayed maturation and extended lifespan, as a coordinated adaptation to a skill‐intensive foraging niche that rewards investment in learning. Skilled foraging permits a longer juvenile period, which permits greater skill, making the account a feedback model as well as a description of a developmental schedule.

*Genetic/neural mutation hypothesis* [57]. Proposes that a relatively late genetic or neural change, around 50–40 ka, enabled fully modern cognition, language and symbolic behavior, producing the Upper Paleolithic transition.

*Learning‐mechanism feedback hypothesis* [38]. Argues that domain‐general learning and memory mechanisms co‐evolved with the accumulating body of cultural information they process, generating feedback that elaborated cognition.

*Provisioning/pair‐bonding hypothesis* [50]. Attributes the origin of bipedality and the distinctive human reproductive pattern to male provisioning and pair‐bonding that permitted shortened interbirth intervals.

*Foraging Niche Hypothesis* [28, 35]. Proposes that entry into a cooperative, information‐intensive hunter‐gatherer foraging niche created the conditions for cumulative culture and the elaboration of human cognition. The account supplies an answer of both kinds—a transition that initiates and a cultural quantity that grows—which is why it appears in two rows of Table 1.

*Sexual‐selection (mating‐mind) hypothesis* [47]. Proposes that mate choice and courtship drove the evolution of human cognitive and creative capacities as fitness displays.

*Cultural brain hypothesis* [41]. Argues that brain size, sociality and life history co‐evolve through the demands of acquiring, storing and transmitting an expanding body of socially learned information. Alone among the informational accounts it specifies population size and connectivity as measurable quantities in the model itself.

*Niche construction theory* [68]. A general evolutionary framework in which organisms modify their own selective environments, generating feedback between organism and environment. It is used here to identify the kind of cause capable of posing a selective problem that returns in intensified form, and not as a replacement for Tinbergen's scheme.

*Cognitive‐niche hypothesis* [34]. Holds that humans occupy a cognitive niche based on reasoning, language and cooperation, allowing them to outwit other organisms and solve problems by mental modeling.

*Variability selection hypothesis* [60, 61, 80, 81]. Proposes that the rising amplitude of Pleistocene climate oscillation selected for behavioral and ecological flexibility rather than for habitat‐specific adaptation. It is the leading candidate whose driver is exogenous to the lineage.

*Hunting hypothesis, “Man the Hunter”* [55, 56]. The classic account in which hunting and meat‐eating drove human intelligence, tool use, cooperation, the division of labor, and social organization.

*Cumulative culture and social ratcheting* [28, 39, 40]. Treats the progressive accumulation and faithful transmission of socially learned innovations as a driver of cognitive change. It is evaluated as a candidate gradient in Section 4.3 and placed in Section 4.4 as an amplifier of directional change rather than a source of its direction.

*Turnover pulse hypothesis* [54]. Holds that pulses of climate change drove synchronized bursts of speciation and extinction across mammalian lineages, including hominins, at discrete intervals.

*Cooking hypothesis* [31, 32]. Proposes that the control of fire and the cooking of food increased dietary energy availability, enabling brain expansion together with reductions in gut and tooth size.

### 3.5 Threshold Accounts, Feedback Accounts, and the Function Slot

Two kinds of account occupy the function slot, and they stand in different relations to the incompleteness identified in Section 2.2.

Threshold accounts identify a one‐off event or transition that initiated divergence, and they cannot on their own generate continuous directional change downstream of the threshold. The point is structural rather than empirical. The savanna hypothesis is the cleanest example. It identifies a late‐Miocene environmental transition as the trigger of hominin divergence and lies wholly outside the relevant interval. Suppose it is correct. The transition is a step function: forest gives way to grassland, the ancestral population encounters a new selective regime, and the lineage adapts. Once the new equilibrium is reached the threshold no longer poses a problem. To explain why the lineage continued to change directionally for two million years afterwards, the hypothesis must invoke either continuing and directional climate change, which would be a different ultimate, or a downstream consequence of the original divergence, which is a mechanism. The same applies to the Turnover Pulse Hypothesis, which multiplies thresholds but leaves the intervals between pulses unexplained, and to Embodied Capital Theory's initial‐niche claim, which accounts for entry into a skill‐intensive foraging niche but not for the subsequent elaboration of technologies and cognitive capacities.

Feedback accounts are different. The Social Brain Hypothesis [42, 43, 44], the Machiavellian‐intelligence hypothesis [45], the Cultural Brain Hypothesis [41], the sexual‐selection account of human cognition [47], the learning‐mechanism feedback of Lotem et al. [38], and the cumulative‐culture accounts of Boyd et al. [40], Sterelny [39] and Vinicius et al. [28] each posit that an evolved change generates selection for further change in the same dimension. They supply function answers of the re‐posing kind and are candidates for the gradient subcategory rather than threshold accounts. Table 1 enters them and Section 4 evaluates them; in the case of Vinicius and colleagues it enters them in the threshold column as well, for the reason given in Section 2.1.

Self‐reinforcing mechanisms are not the problem. The prey‐size‐decline account is itself a runaway loop, in which predation depletes the resource base that then selects for more efficient predation, and Fisherian runaway is formally specified and empirically supported in sexual selection [76, 77]. What distinguishes candidates within this family is what carries the loop. Every feedback loop requires something that holds the effect of one generation's activity so that the next begins from an altered state; in niche‐construction terms that is the ecological inheritance, and here it is the loop's carrier. Four properties of the carrier matter: whether it is internal to the lineage or lodged in the world outside it; whether the altered state persists once the activity that produced it relaxes; whether it is bounded by allocation or by drawdown of a slowly renewing resource; and whether its state can be read from evidence independent of the response it is invoked to explain.

The fourth property follows from the first. A carrier held in bodies is characteristically inferred from the very trait it is invoked to explain: the intensity of social selection from the neocortex ratio, the skill demand of a foraging niche from the technology used to meet it. A carrier lodged in the environment can in principle be read from evidence that owes nothing to the response. A loop carried internally, regenerated each generation and bounded by allocation, is not the kind of thing that can pose a problem of continuously rising magnitude across two million years—and a loop whose carrier cannot be read independently cannot be shown to have done so even if it did.

Cultural evolutionary processes can generate persistent directionality through path dependence, recombination, an expanding division of labor, competition among groups, prestige and payoff biases, institutional accumulation, and change in the size and connectivity of learning populations. Once cultural capacities alter diets, mortality, cooperation and population density, they become part of the selective environment, which is niche construction in the sense used here. Cultural feedback belongs in the gradient subcategory and is evaluated there like any other candidate.

The major hypotheses of human evolution can therefore be classified by which Tinbergen question they answer and, within the function question, by whether the answer is threshold‐like or re‐posing. The table records each hypothesis's stated driving factor rather than the totality of what it contains; entries in the gradient column are candidates, not accepted occupants.

Once a re‐posing problem has been identified, the remaining question is causal. Not every feedback loop can generate rising magnitude over evolutionary time, and not every directional pressure can be measured independently of the traits it is invoked to explain. The threshold‐gradient distinction therefore needs criteria that separate structurally possible gradient causes from labels that merely describe escalation.

## 4 Criteria for a Gradient Ultimate Cause

### 4.1 Definition of a Gradient

A selective problem may be posed once and cease, recur at constant magnitude, or recur at rising magnitude. The first is a threshold; the third is a gradient. The second is the ordinary condition of most traits in most lineages, including Tinbergen's own examples, and produces stasis rather than change.

Both terms name kinds of selective problem rather than kinds of environmental agent. The problem is that prey are becoming smaller and harder to catch, not simply that prey‐size decline caused encephalization. Keeping the formulation inside Tinbergen's function question preserves the distinction between what a trait contributes to survival and reproduction and the historical cause of a trend. Function, ultimate cause, selective problem, causal driver and carrier are related but not interchangeable: the criteria apply to selective problems and ultimate causes; the source criterion asks whether the driver is endogenous or exogenous; and the carrier is the state that stores the effect of one generation's activity for the next.

A gradient need not produce a smooth response. The pressure rises progressively, but the trait may respond episodically, making plateaus compatible with the category. In quantitative genetics a selection gradient is the partial regression of relative fitness on a trait; a gradient cause in the present sense sustains a non‐zero selection gradient in the same direction over evolutionary time.

The constant‐magnitude case matters because it accounts for plateaus. The same apparatus that identifies a gradient also identifies intervals in which no gradient is operating, so alternation between directional phases and stasis becomes a prediction rather than accommodation.

Three established concepts are easily conflated with gradient causes, and each misses a different part of the problem. Sustained directional selection names the outcome, gene‐culture coevolution names one way change can be transmitted and amplified, and runaway selection names a feedback form. None by itself identifies the source of a directional, measurable, cross‐trait problem of rising magnitude.

Sustained directional selection names an outcome rather than its source. Selection can be held in one direction by continuous external forcing or by an internal feedback loop, and the gradient category exists to ask which. A gradient is one kind of answer to the question of what sustains sustained directional selection, not a synonym for it.

Gene‐culture coevolution specifies how change is transmitted, retained and amplified across generations, and it can operate under any of the three relations described above. It answers the mechanism question in Tinbergen's scheme. It becomes a candidate gradient only if the cultural term itself carries a directional asymmetry, which is what the asymmetry half of the temporal‐course question asks, and cultural transmission is compatible with a trajectory running in either direction or in neither.

Runaway selection is a feedback loop with positive gain, and every NCT‐style gradient is a runaway loop of this kind. The converse does not hold. Runaway explains escalation without explaining the choice of direction: Fisher's model is neutrally stable along a line of equilibria, so direction depends on initial conditions and is, in the general case, arbitrary and lineage‐idiosyncratic [76, 77]. A gradient requires an asymmetry that fixes direction independently of initial conditions. The temporary course and source criteria discriminate among candidates on this point.

### 4.2 Interval of Application

The relevant interval is roughly the past two million years. The lower bound follows from two independent considerations. Physiologically, the obligate‐carnivory niche, meaning a dietary regime in which animal‐sourced food is required rather than facultative in meeting the energy and nutrient budget (Ben‐Dor and Barkai [64]), is a feature of *Homo* rather than of earlier hominins, and the protein and fat requirements it imposes do not apply before. Statistically, Gingerich [14] finds hominin endocranial volume in relative stasis from 3.2 to 2.0 Ma and directional thereafter. A physiological argument and a statistical one converge on the same boundary.

### 4.3 Rules for Comparison

Each hypothesis is evaluated on its own stated premises: Dunbar's rising group sizes, Muthukrishna et al.'s accumulated transmitted knowledge, Kaplan and colleagues skill‐rewarding resource base, Potts's increasing amplitude of climate oscillation, and prey‐size decline as driven by hominin predation. The truth of those premises is a separate empirical matter, settled case by case against the archeological, paleontological and ecological records, and for our own premise it is argued elsewhere [64, 65] rather than here. The structural question is whether the mechanism each hypothesis specifies, taken exactly as its authors specify it, can supply a re‐posing function answer.

This rule makes the comparison symmetric and fixes the force of the verdicts. If variability selection fails the source criterion, the claim is not that Potts is wrong about Pleistocene climate; it is that the causal driver he identifies is exogenous to hominin activity. A hypothesis may satisfy every criterion and still be false, and it may fail a criterion while remaining valuable for another explanatory role.

### 4.4 The Criteria

A complete gradient explanation must answer five questions. They are elements of the explanation rather than pass‐fail tests, and a candidate may supply one part of a question while leaving another unanswered.

#### 4.4.1 Temporal Course

Over what interval did the problem recur, and what made its change one‐way? A gradient must operate across the interval it is invoked to explain rather than in only one phase. Continuous operation does not require constant intensity; it requires that the mechanism generating the pressure remains active rather than switching on and off. A driver may operate while its effect is masked by an oscillating term, so plateaus may include intervals of this kind.

Chained or superimposed drivers are possible. Hominin evolution may have been driven by one factor early and another later, or by several at once. A chain carries two burdens a single asymmetric driver does not: it must explain the timing of each handover and why direction was preserved across it. Two successive drivers produce a continuous trend only if they push the same way. The criterion is therefore a parsimony argument rather than a prohibition.

Operating throughout is not sufficient: a gradient must also produce net displacement, not merely change. A driver whose steps average to zero produces no trajectory however large those steps are and however much they grow, so a gradient requires a non‐zero mean step, and that requires an asymmetry in the generating process. The cleanest such asymmetries are irreversibilities: extinctions, depletions of resources that renew more slowly than they are removed, and irreversible thermodynamic transitions accumulate because their steps run one way. Displacement is assessed over the interval as a whole. A sub‐interval whose mean step cannot be distinguished from zero is predicted rather than tolerated; what would fail is a mean step indistinguishable from zero across the whole.

Monotonicity, as defined in Section 1.1, is separate from directionality. Hominin trait series are expected to be directional and only moderately monotonic; a strictly monotonic series would count against the prey‐size‐decline account.

#### 4.4.2 Source

Temporal course asks whether a problem recurred in one direction; source asks what made that recurrence endogenous or exogenous. Under the NCT extension, a gradient is driven by niche‐constructing activity rather than by forcing outside the lineage. The organism's response must modify the environment in ways that intensify the pressure for subsequent generations. A feedback loop running entirely within the organism does not satisfy this requirement. Embodied Capital Theory, in the gradual reading, is a genuine feedback model in which skilled foraging permits a longer juvenile period which permits greater skill, but nothing in that circuit alters the resource base, and the model converges on an interior optimum for a given skill‐return function [78]. Such cases are internal feedback rather than environment‐modifying loops. An internal answer is not wrong for that reason; it supplies no environmental term whose change can be described.

Endogeneity is sufficient but not strictly necessary. Genuinely continuous and directional exogenous forcing could in principle qualify, so the leading exogenous candidate is evaluated rather than excluded by definition.

#### 4.4.3 Scope

Which lineages faced the problem, at what level, and which traits does it demand? A gradient generated by NCT‐style feedback acts primarily on lineages engaged in the relevant niche‐constructing activity, which explains why hominins might follow a directional trajectory while sympatric lineages did not. An exogenous gradient would push co‐occurring lineages in the same direction, and differential responses would then have to be attributed to lineage‐specific traits, returning the explanation to the mechanism slot. A gradient also predicts congruent effects at two levels: directional change within lineages that track the pressure, and differential persistence among those that track it well. These are consequences of one cause rather than competing explanations.

The second half of the question concerns cross‐trait scope. A gradient must coherently predict the cluster of traits that changed rather than a single trait. The claim is functional rather than statistical: one adaptive problem can drive several response systems with different costs, constraints and lags, producing offset and nonlockstep change rather than tight covariance.

#### 4.4.4 Measurement

By what evidence is the driver established independently of the response it explains? A gradient's driving variable must be measurable in the record without being inferred from the traits whose change it explains. When group size is estimated from neocortex ratio [42], or the skill‐intensity of a resource base from the technology used to meet it [48], the loop lacks an external carrier: coupling cannot be estimated, lag structure cannot be tested, and correlated change cannot be demonstrated without circularity. This question differs from scope. A loop may be endogenous and unmeasurable, as in the Machiavellian account, while a causal driver may be exogenous and well measured, as climate variability is from isotope and dust‐flux series. The requirement is demanding and is not met uniformly, including by our own account.

#### 4.4.5 Budget

What paid for the response, and how did payment remain possible as the response accumulated? The question follows from the cost argument in 2.2: a constant benefit set against a cost that does not fall yields an optimum, and selection at an optimum produces stasis. Sustained change requires the return on a further increment to keep outrunning what the increment costs. An account that does not state what paid for the response has not shown that the response was affordable.

The requirement is not limited to metabolism, since time, risk and foregone alternatives are budgets too, but metabolism is central because every candidate considered here is ultimately an account of brain expansion, and encephalization is among the most energetically expensive adaptations in mammalian biology [29, 30]. An account may answer by identifying a new or enlarged energy source, by specifying a reallocation that frees the necessary budget, or by showing that the marginal cost falls. It may not leave the question unasked.

The budget question is not specific to gradients. Any explanation of an expensive trait change is open to it. For most traits it is answered trivially, because most traits cost little, and a trait that costs nothing and confers nothing is invisible to selection and will drift. The question becomes substantive when a trait is expensive. Its absence from the four‐question scheme is visible in how the field handles encephalization: the expensive‐tissue hypothesis [29] and the expensive‐brain framework [30] ask what paid for brain expansion, yet Table 1 places both under mechanism because there is nowhere else to put them. Neither is an account of how the brain works. Survival value asks what a trait contributes and treats cost as one pressure in the same ledger; it does not ask what resources were available to meet that cost.

### 4.5 Limits of the Criteria

The criteria are necessary rather than sufficient. A candidate satisfying all five remains subject to empirical comparison with alternatives; the criteria establish only that it is structurally capable of doing the work required of a gradient cause.

Several findings would count against the prey‐size‐decline account. If a directional model does not outperform an unbiased random walk for the hominin endocranial series at 100‐kyr resolution across the interval, the claim of a sustained gradient is unsupported. If mean prey body mass cannot be separated in the faunal record from climate‐driven faunal restructuring, the measurement criterion is not met. If regional sequences show the predicted technological change without antecedent change in prey‐size distribution, the specified loop is not the driver.

The criteria make the threshold‐gradient distinction comparative. A feedback account may qualify as a candidate gradient and still fail if it lacks direction, an external carrier, cross‐trait scope, independent measurement, or a budget. The existing candidates can therefore be compared structurally before their empirical premises are adjudicated.

## 5 Evaluating the Current Occupants of the Gradient Subcategory

**Figure 3:**
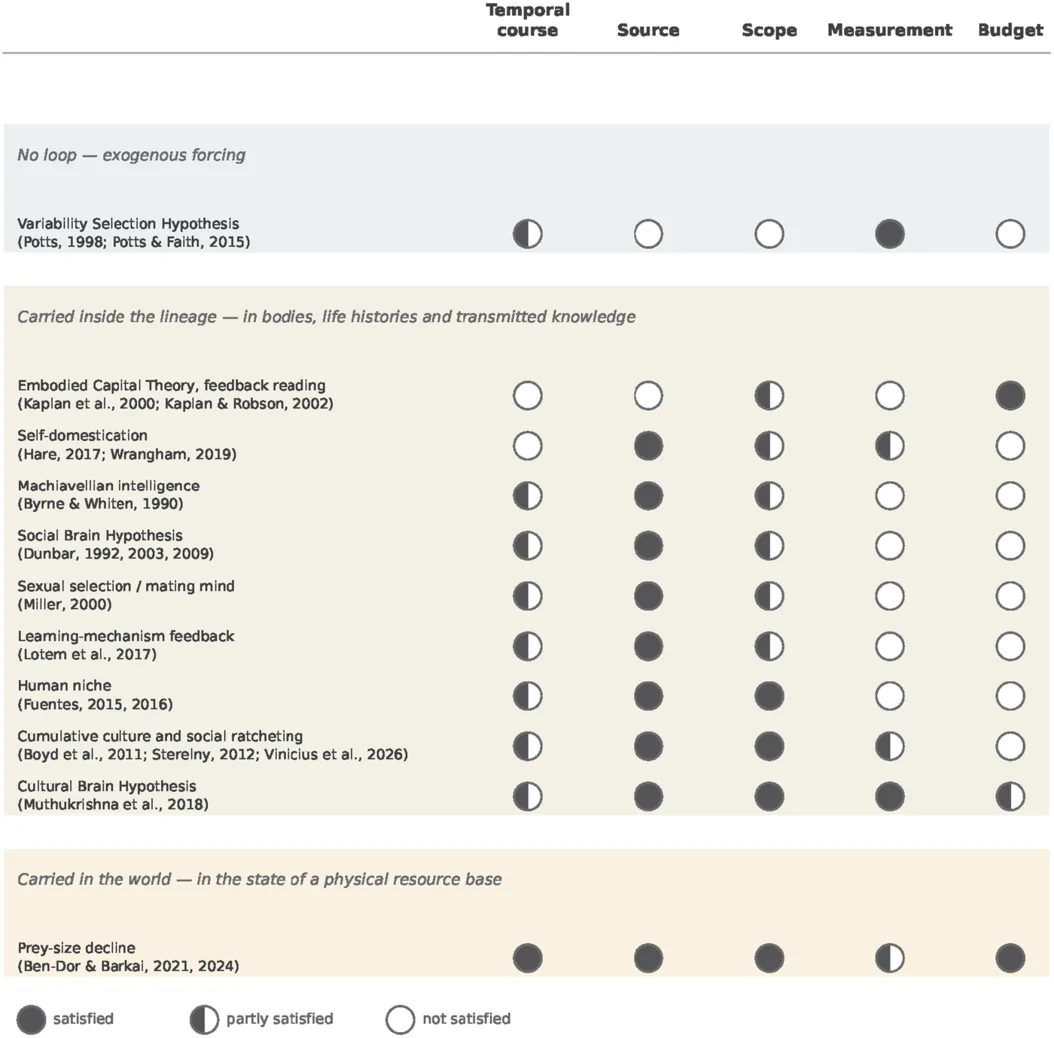
Candidate gradients against the five questions. The five questions are named across the top; two have two parts, labeled beneath, so that partial answers remain visible. Rows are grouped by what carries the loop: no loop, a carrier internal to the lineage, and a carrier in the physical world. A filled circle indicates that a candidate supplies the element, a half‐filled circle that it supplies it partly, and an open circle that it does not. Within each group candidates are ordered by how many elements they supply; ties are not significant. Verdicts record what each account states on its own premises rather than whether those statements are correct.

Several hypotheses occupy the gradient sub‐category even though they have often been read as threshold or mechanism accounts. They differ chiefly in what carries the loop: no loop, an internal allocation schedule, transmitted information, or a state of the physical world. Figure 3 records how each candidate meets the five questions. Candidates within a family fail, where they fail, for the same structural reason.

### 5.1 Exogenous Gradients: The Variability Selection Hypothesis

The variability selection hypothesis [60, 79] is the only major framework in human‐evolution research that has explicitly attempted to identify a directional environmental driver operating across the long Plio‐Pleistocene span rather than at a single threshold. Potts argued that the rising amplitude of climatic oscillation through the Cenozoic generated growing inconsistencies in selection, favoring plasticity rather than habitat‐specific adaptation. Potts and Faith [61] formalized eight high‐variability stages across the past 5 Myr, and the Olorgesailie drilling project [80, 81] tied a sharp variability rise at 400–320 ka to the Acheulean–Middle Stone Age transition.

Climate variability is a real signal, well documented in oxygen‐isotope and dust‐flux series. Potts's stage scheme usefully identifies intervals of intensified variability, and the Olorgesailie papers establish a correlation between variability, faunal turnover and behavioral change at one well‐dated locality. Potts and colleagues may not have intended the hypothesis to account for the full trajectory; we test it because it is the leading exogenous candidate, not to fault it on its own terms.

On temporal course it answers half the question and fails the other half. The stages sit in the Plio‐ and early to Middle Pleistocene, leaving the Upper Paleolithic transition, the broad‐spectrum revolution and the Neolithic outside the framework as elaborated. More fundamentally, variability is a property of variance rather than mean. High variability selects for adaptability, which widens a reaction norm rather than displacing it, and Potts's stages alternate between high‐ and low‐variability windows while brain size, technology, range and cooperation do not alternate. Extracting a directional trajectory from nondirectional forcing requires an additional asymmetry, which Potts implicitly supplies through cultural transmission—the same machinery used by informational accounts. Without that ratchet, the hypothesis predicts oscillation in plasticity rather than accumulation.

On source it is exogenous by its own specification: variability is driven by orbital, oceanographic and tectonic processes independent of hominin activity, and no loop returns the response to the variability the next generation faces. On scope, Pleistocene variability was experienced by every African mammal lineage, several of which show plasticity responses while none ratcheted to anything resembling the hominin trajectory. Potts appeals to lineage‐specific starting traits such as bipedality and omnivory, but that relocates the explanatory work: if those traits decide who responds and how, variability is a permissive context rather than a directional driver. Nor does an oscillating climate demand that lithic elements become smaller and more composite, that ranges expand in one direction, or that life history slow. Coping with variability explains a generalized increase in buffering capacity, not the specific and co‐directional character of the package.

It has no answer to the budget question: an increase in environmental variability identifies no source of energy for a more expensive brain, and adaptability is itself costly. On measurement, by contrast, it does well, and better than our own candidate does for some traits. Climate variability is recovered from isotope and dust‐flux series entirely independent of the hominin record, so the driving variable is established without presupposing the response. This is the one question the hypothesis answers cleanly, and the objection set out above is unaffected by it: an account whose driver is impeccably measured still cannot produce net displacement if what is measured is a variance.

### 5.2 Allocation Gradients: Embodied Capital Theory

The contrast with Embodied Capital Theory is instructive because its weakness lies in the opposite place. Variability selection has an external driver but no endogenous loop; Embodied Capital Theory has a real feedback loop but no environmental carrier. Skilled foraging permits a longer juvenile period, which permits greater skill, and lower extrinsic mortality raises the return on investment in embodied capital, a circuit Kaplan and Robson [78] formalize. It is nonetheless equilibrating rather than self‐amplifying. For a given skill‐return function it converges on an optimal allocation between growth, learning and reproduction, and once there the pressure is spent. Sustaining it requires the skill‐return function itself to keep shifting, and the model does not say why it should.

The circuit carries no environmental term. In every other feedback account here the environment that selects is constructed by the organisms, whether group size, a transmitted repertoire or a distribution of mating preferences. Here the resource base is unchanged by the response, and the loop runs inside life‐history allocation, leaving the source question unanswered. A reading in which foraging returns rise as knowledge expands supplies the missing term, but then the theory becomes cumulative culture and inherits the reversibility problem of 4.3. On budget, however, Embodied Capital Theory does better than any other candidate in this section: it is an energetic allocation model and specifying how investment in embodied capital is funded is exactly what it does well. It answers the budget question and not the source question.

### 5.3 Informational Gradients

Informational gradients supply what allocation gradients lack: an environment constructed by the organisms themselves. The Social Brain and Machiavellian‐intelligence hypotheses, the Cultural Brain Hypothesis, learning‐mechanism feedback, self‐domestication, the human‐niche account and the cumulative‐culture accounts share a structure: an evolved capacity generates a body of socially transmitted or socially constituted information, and that body selects for further capacity. They answer the source question without difficulty, since the environment that selects is genuinely constructed by the organisms, and they answer the first half of the scope question as a consequence.

They answer the first half of the temporal course question and not the second, and they fail it together. The asymmetry that produces a nonzero mean step must reside somewhere, and in these accounts, it resides in the persistence of transmitted information. That such information is reversible is not by itself the objection, since the definition adopted in 1.1 permits reversals: a process may lose repertoire locally and still carry a biased expectation. The objection concerns what the bias depends on. In these models, accumulation exceeds loss when populations are large, dense and well connected, and fail when they are not. This is a dependence the Cultural Brain Hypothesis makes explicit by placing population size and connectivity in the model itself. Direction is therefore set by demographic and connectivity conditions rather than generated within the informational loop, and those conditions are not explained by it. An informational ratchet accordingly predicts the plateaus and local reversals the record shows, which is a point in its favor. What it does not supply is a reason internal to the loop why the rate of gain should exceed the rate of loss across two million years, which is why 4.4 places these accounts as amplifiers of directional change rather than as its source.

They fail on measurement for a reason that follows from where their carrier sits: a carrier held in bodies and transmitted information is characteristically inferred from the trait it is invoked to explain. Group size in the Social Brain Hypothesis is estimated from the neocortex ratio; Machiavellian intelligence has no archeological proxy for the intensity of social manipulation. The Cultural Brain Hypothesis fares better, since population size and connectivity are in principle measurable from genetic and archeological evidence. That is the version with a genuine external carrier, and it answers the measurement question where the others do not.

Vinicius et al. [28] identify circularity between culture and cognition as the central problem for any cumulative‐culture account and answer it with a timing argument: dependence on material culture precedes cognitive change, since hominins with ape‐sized endocranial volumes were making stone tools by 3.3 Ma. The answer secures their threshold. It does not carry over to their gradient. Once the driving variable becomes the growing body of accumulated culture, its magnitude is read from the archeological record of technology, which is the response that growing intelligence is invoked to explain. The circularity close at the origin reopens along the trajectory, showing why threshold and gradient answers carry different burdens.

They also fail on budget. Informational loops explain how cultural repertoire propagated and were retained; they do not supply the energy that paid for the organ doing the retaining. The Cultural Brain Hypothesis is the partial exception, since it models the cost of brain tissue explicitly, but modeling a cost is not the same as identifying what met it. Informational accounts are generally offered as operating throughout hominin evolution, and on their own premises they answer the first half of the temporal‐course question. What they do not supply is the second half: a nonreversible source of direction.

The three failures are one failure. A loop rebuilt each generation out of the trait it explains has nothing within it to enforce direction, nothing outside it to be read against, and nothing in it to pay. Embodied Capital Theory shows the same point from the other side: carried inside the lineage but running on allocation, it answers the budget question well and fails the source question for the same reason, since energy moved within a fixed envelope says what paid rather than what changed. The criteria divide this family internally rather than treating all feedback accounts alike.

### 5.4 Amplifiers Rather Than Gradients

Neither variability selection nor cumulative culture satisfies the five questions, and neither is therefore mistaken about the phenomenon it identifies. Climate variability is real and intensified‐variability windows correlate with behavioral change; cumulative culture is real and retains and propagates innovations. Both operate as amplifiers of selective change rather than as sources of its direction: adaptability equips a lineage to endure fluctuation, and transmission propagates whatever responses arise. Both belong in an integrated account, and heightened adaptability may have furnished the flexibility that allowed hominins to track a changing resource base at all. Variability selection is therefore not a rival to a gradient hypothesis but a facilitator of the adaptations a gradient selects for.

The failures in Section 4 are diagnostic rather than eliminative. They specify what a successful candidate must supply: a directional carrier outside the response, cross‐trait reach, independent measurement, and a budget for an expensive organ. Prey‐size decline is useful because, if its premise is granted, those elements occur in one loop.

## 6 Prey‐Size Decline as a Worked Example

### 6.1 Premise and Status of the Example

Prey‐size decline is the example we work through; its structure can be assessed independently of whether its empirical premise is ultimately accepted. Its detailed outline and tests are presented elsewhere [64, 65, 82], and the threshold‐gradient framework does not depend on it.

The premise is that hominins became obligatory specialized predators of large‐bodied prey during the interval, and that hominin predation of the largest available prey was a principal driver of the decline in mean prey body size. Granted that premise, the causal structure is a niche‐construction loop rather than a threshold event or an internal informational ratchet.

### 6.2 The Loop

The mechanism is a niche‐construction loop with a physical rather than an informational ratchet. Hominin predation falls disproportionately on the largest available prey; the largest prey populations decline or go extinct, in subsequent generations selection on brain size, weapons, cooperation, ranging and other traits rises, because smaller, faster, more dispersed and more evasive prey cost more per unit of energetic return; improved predatory efficiency deepens the depletion the next generation faces, and the loop iterates.

Direction is set by an ecological asymmetry with prey demographic source. The traits that allow prey populations to absorb predation, early maturity, short gestation, large and frequent litters, high population density, all scale inversely with body mass, so recruitment at the large end of the prey distribution is low relative to the offtake a predator can impose. Cardillo et al. [83] show that this is not a marginal effect. Above a body mass of roughly 3 kg, extinction risk in mammals is generated by intrinsic life‐history traits acting together with external pressure; below that threshold, external pressure alone accounts for it. Every prey animal at issue here lies far above that threshold.

The intrinsic trait supplies the vulnerability, hominin predation supplies the external pressure, and neither alone would produce the outcome.

The loop has two terms. A directional depletion term operates alongside an oscillating climatic term that affects prey abundance and body‐size distribution independently of predation. Where the climatic term is momentarily larger and favorable to large prey recruitment, the trajectory can pause or reverse locally without the gradient having failed. The result should be plateaus or temporarily reversed rather than strict monotonicity, and intervals of technological stasis should coincide with intervals in which mean prey body mass is stationary.

### 6.3 The Candidate Against the Criteria

The premise stated in Section 5.1 is granted throughout what follows. What is assessed is the structure of the account that premise generates, and not the evidence for the premise itself, exactly as in Section 4.

#### 6.3.1 Temporal Course

The problem recurs across the whole of the interval rather than in one part of it, and it does so by construction: each generation of predators faces a prey distribution already shifted by its predecessors, so the pressure does not switch on at a particular date and does not require one.

The change is one‐way for the demographic reason given in 5.2, and the claim should be stated at the right strength. It is not that the distribution never recovers. Locally and over short spans it does: populations re‐expand, ranges shift back, and the climatic term of 5.2 can dominate the depletion term for a time. What the demography establishes is that replacement at the large end is slower than removal there, so recoveries do not cancel the losses and the expected step across the interval remains negative. Irreversibility in the sense the criterion requires is an asymmetry of rates, not an absence of reversals, and that is what supplies the nonzero mean step.

This is the structural difference from the informational gradients of Section 4. Their asymmetry resides in transmitted information, where nothing holds the rate of loss below the rate of gain, so the ratchet has to be assumed; here it follows from what carries the loop.

#### 6.3.2 Source

The response modifies the environment that selects. Predation concentrated at the large end of the prey distribution alters that distribution, and the response it selects for, greater efficiency against the size classes that are still available, feeds back into the same distribution, so what a later generation confronts is partly the product of what earlier ones did. The loop is therefore environment‐modifying rather than internal, and the carrier is lodged in the world, in the state of a physical resource base, rather than in bodies or in transmitted information.

#### 6.3.3 Scope

The loop is entered only by a lineage that takes the largest available prey preferentially. A consumer whose offtake is not concentrated at the large end of the distribution does not deplete it and so does not generate the pressure, which makes the account lineage‐specific by construction rather than by stipulation. It follows that the account predicts congruent effects at two levels: directional change within the lineages that entered the loop, and differential persistence among those that entered it less deeply.

On the second half of the question, the need to take progressively smaller, faster, and more dispersed prey plausibly demands the cluster at once: lighter and more diversified weapons, more components per implement, more selective raw material carried further, expanded foraging ranges, more elaborate tracking and decision‐making, slower life history to support the required learning, and more cooperative organization. Each of these improves predatory efficiency on the next smaller size class, which is what couples the response back to the driver rather than leaving it a list of consequences. The cluster also contains one member the alternatives do not entail: the accounts evaluated in Section 4 require technology to become more complex, and none of them requires its elements to become smaller.

#### 6.3.4 Measurement

The driving variable is mean prey body mass, and it is recovered from faunal assemblages by evidence that owes nothing to endocranial volume or to lithic attributes. That independence is what makes the coupling estimable and the lag structure testable, and it is precisely what the internally carried accounts of Section 4 lack.

Independence is partial, however, and the limit should be stated. An archaeofaunal assemblage records what was taken and carried to a site rather than what was available on the landscape, so it is shaped by prey choice, transport and site function, that is, by the response as well as by the driver. Measuring the variable as a within‐assemblage composition rather than as a count reduces exposure to differential preservation but does not remove the behavioral component. What answers the question fully is the background faunal community, recovered from paleontological assemblages that owe nothing to hominin site formation; where both are available the difference between them is the record of selectivity rather than a confound, and where only archaeofauna exist the driver is measured less independently than the criterion requires.

Whether the measurements in fact bear the account out is a separate matter, and not one settled here. The question is also not answered for cooperative organization, which has no independent quantitative proxy, and we record that as a limitation rather than resolving it.

#### 6.3.5 Budget

The loop supplies its own budget, and this is where it differs most sharply from the informational accounts. The resource whose depletion generates the pressure is the same resource whose consumption paid for the response, since large‐bodied prey are not merely larger packages but disproportionately fatter ones. The account therefore carries a tension it should acknowledge rather than conceal: the resource that pays is the resource being depleted. Improved efficiency on smaller prey is what keeps the budget solvent as the large end of the distribution disappears, and the loop ends, as 5.4 sets out, when the depleted prey base and the metabolic ceiling are reached together. Source and budget are distinct questions that happen to share an answer here. In an exogenous account they do not, since the source of the pressure and the source of the payment are then unrelated. That one variable answer both is a property of a loop carried in a consumable resource.

### 6.4 How the Loop Ends

Two endpoints complete the account without requiring a separate explanation. Brain expansion, the most metabolically expensive response [84], approaches a ceiling with the evolution of H. sapiens [85] and has declined thereafter, since the terminal Pleistocene [4]. When the depleted prey base and the metabolic ceiling are reached together, the loop resolves not in equilibrium but in niche replacement, as the lineage abandons large‐prey predation for food production and initiates a new niche‐construction loop [71]. A gradient that specifies its own termination condition is more constrained than one that does not, and the informational accounts of Section 4.3 do not specify one.

## 7 Integration, Predictions, and Conclusions

### 7.1 The Integrated Account

With the candidate gradient in place, the other hypotheses fall into complementary roles, and the places are not in competition. The function‐threshold subcategory is occupied by the savanna and comparable initial‐niche transitions, which lie at or before the start of the interval. The function‐gradient subcategory is occupied by the candidates evaluated in Section 4, of which the informational ratchets fail the temporal‐course, measurement and budget questions, Embodied Capital Theory fails temporal course and source, variability selection answers only the measurement question in full, and prey‐size decline is offered as a candidate answering four in full and one partially. The mechanism slot is occupied by the energetic accounts [29, 30, 31], by endurance running [33], and by cumulative culture and social ratcheting [28] in their capacity as transmission machinery. The ontogeny slot is occupied by Embodied Capital Theory [48] and the Grandmother Hypothesis [49]. The phylogeny slot is occupied by comparative analyses [62, 63]. Prey‐size decline is one additional candidate for the gradient role rather than a replacement for the accounts occupying the other slots.

### 7.2 Predictions and Tests

As in Section 1.1, directionality does not mean smooth monotonic change. The tests therefore look for biased displacement over the interval, coupled with reversals, pauses and plateaus where an oscillating term temporarily masks the directional one.

Discriminating tests require measures specified in advance. The strongest predictions concern directional mode, temporal precedence, covariation of rates, plateau coincidence, comparative absence, termination, discrimination from alternatives, and taphonomic robustness.

#### 7.2.1 Directional Mode

Fitting stasis, unbiased random walk and directional models to hominin trait series at 100‐kyr resolution [5] should favor the directional model across the interval for endocranial volume and for mean lithic element size. Kendall's tau should differ significantly from zero in both cases, with magnitude well below one and with the sign the account predicts: positive for endocranial volume, negative for element size. A best‐supported random walk would count against the account, as would a tau near ±1, and so would a sign opposite to the one predicted. Maximum transfer distance is excepted, for the reason given in 1.4.

#### 7.2.2 Antecedence

In regional sequences with adequate faunal and lithic resolution, change in the prey‐size distribution should precede the predicted technological and mobility response. Both variables must be dated independently, and the test should be run in both directions: technological change of the predicted kind without antecedent prey‐size change would count against the loop.

#### 7.2.3 Covariation of Rates

The rate of decline in mean prey body mass and the rate of change in toolkit composition should covary regionally. Toolkit composition should be measured by procedural units [7] and by techno‐units [9] rather than by type counts, which are sensitive to taxonomic practice and to sample size.

#### 7.2.4 Plateau Coincidence

Intervals of technological stasis should coincide with intervals in which mean prey body mass is stationary. The Acheulean stasis between roughly 1.5 and 0.7 Ma, which coincides with Gingerich's second endocranial stasis phase, is the primary case. No informational‐ratchet account predicts this coincidence.

#### 7.2.5 Comparative Absence

Sympatric nonhominin carnivore lineages, which did not preferentially deplete the largest prey, should show no comparable directional trajectory over the same interval.

#### 7.2.6 Termination

Agricultural origins should cluster temporally with local large‐prey depletion rather than with climate stabilization alone, and stable‐isotope series should show declining megaherbivore consumption toward the terminal Pleistocene.

#### 7.2.7 Discrimination from Alternatives

The predictions must be tested against demographic and climatic alternatives rather than against a null of no pattern. Where prey‐size decline and climate variability are confounded, as at Olorgesailie, discrimination requires localities where they are not. In the 400–320 ka window at Olorgesailie, a sharp rise in climate variability, roughly 85% turnover in the mammalian community, and the replacement of the Acheulean by an early Middle Stone Age assemblage are concurrent [80, 81], so a variability reading and a prey‐size reading predict the same evidence there. Decisive cases require prey‐size decline without a variability rise, or the reverse.

#### 7.2.8 Taphonomic and Sampling Effects

Taphonomic and sampling effects require treatment in every case. Counts of tool types and site types are inflated by the improving quality of the record toward the present. Mean prey body mass, measured as a within‐assemblage composition, is less exposed to this than the response variables it is invoked to explain.

### 7.3 Conclusion

Tinbergen's scheme is not the obstacle to explaining directionality in human evolution. It is the instrument, but only if the function question is allowed to detect two kinds of incompleteness. Asked once of a trait, it cannot reveal that its answer must be re‐given; asked trait by trait, it cannot reveal whether the answers given for different traits are one answer or several. Much of the field consists of answers to a question asked once, and the difference between one answer and several is the difference between a single explanation of the hominin trajectory and a coincidence of separate ones.

The two additions are modest in form and consequential in effect. They generate the threshold‐gradient distinction from within the scheme, supply a conditional and testable completeness standard, and make it possible to ask not only whether a hypothesis is true but what kind of explanatory work it could do if true. Niche Construction Theory supplies the kind of cause a gradient requires. The five criteria make candidates comparable. Prey‐size decline is one candidate worked through those criteria, conditional on a premise argued elsewhere.

Crucially, the value of this extended Tinbergen scaffold is entirely independent of whether the prey‐size decline hypothesis is ultimately accepted. Its core demand is more general: any explanation of human evolution must state which Tinbergen question it answers, whether it explains the appearance of a trait or the magnitude that trait reached, and whether the answer given for one trait also serves the others. Those three answers determine how much of the trajectory remains unexplained. The budget question is broader still: an account of any expensive trait that does not say what paid for it is incomplete in the same way, and Tinbergen's scheme as it stands offers nowhere to record the answer. The missing entry is not another question, but a way of recognizing when the function question must be asked again.

## Data Availability

The authors have nothing to report.
